# B-Cell Targeted Therapies in Patients with Multiple Sclerosis and Incidence of Headache: A Systematic Review and Meta-Analysis

**DOI:** 10.3390/jpm12091474

**Published:** 2022-09-08

**Authors:** Theodoros Mavridis, Nikolaos Papagiannakis, Marianthi Breza, Georgios D. Vavougios, Kostas Patas, Ariadne Daponte, Achilleas Laskaratos, Paraschos Archontakis-Barakakis, Ioannis Pantazopoulos, Dimos D. Mitsikostas

**Affiliations:** 1 1st Department of Neurology, Eginition Hospital, Medical School, National and Kapodistrian University of Athens, 72-74 V. Sofia’s Avenue, 11528 Athens, Greece; 2 Neuroimmunology Laboratory, Department of Neurology, Athens Naval Hospital, 11521 Athens, Greece; 3 Department of Respiratory Medicine, Faculty of Medicine, University of Thessaly, Biopolis, 41334 Larissa, Greece; 4 Department of Computer Science and Telecommunications, University of Thessaly, Galaneika, 41334 Lamia, Greece; 5 Department of Nursing, University of Thessaly, 41334 Larissa, Greece; 6 Medical School of Athens, National and Kapodistrian University of Athens, 11527 Athens, Greece; 7 Northeast Internal Medicine Associates, LaGrange, IN 46761, USA; 8 Department of Emergency Medicine, Faculty of Medicine, University of Thessaly, Biopolis, 41500 Larissa, Greece

**Keywords:** B-cell targeted therapies, headache incidence, cladribine, ocrelizumab, ofatumumab, ublituximab, rituximab, purinergic signaling

## Abstract

Background: Multiple Sclerosis treatment with B-cell targeted therapies may be associated with an increased incidence of headache. We aimed to find and compare the association of B-cell targeted therapies with the incidence of headache in patients with Multiple Sclerosis. Methods: In a systematic based approach, the following databases were searched from inception until the 6th of June 2020: Pubmed/MEDLINE, ClinicalTrials.gov, EU Clinical Trials Register. Only randomized clinical trials (RCTs) enrolling patients with Multiple Sclerosis comparing B-cell targeted therapies (Rituximab, Ocrelizumab, Ofatumumab, Ublituximab or Cladribine) with placebo were selected for the systematic review and further meta-analysis. PRISMA guidelines were followed at all stages of the systematic review. The primary outcome was an all-cause headache of B-cell targeting therapy in patients with Multiple Sclerosis. Results: Nine RCTs were included. Compared with placebo, treatment with B-cell targeting therapies revealed a trend in headache risk, but it was not statistically significant (Relative Risk 1.12 [95% Confidence Interval 0.96–1.30]; *p* = 0.15; I^2^ = 9.32%). Surprisingly, in a sub-group analysis, Cladribine was statistically significant for an increase in headache risk (RR 1.20 [95% CI 1.006–1.42]; *p* = 0.042; I^2^ = 0%; 3 studies with 2107 participants). Conclusions: Even though a trend is shown, B-cell targeted therapies do not correlate with an increased incidence of headache as an adverse effect. Sub-analyses revealed a significant association between Cladribine alone and an increased incidence of headache. Whereas a purinergic signaling cascade is proposed as a mechanism of action, further research is needed to unravel the underlying pathogenetic mechanism of headache induction and establish headache prevention strategies.

## 1. Introduction

Headache disorders are ranked as the fourth most common among neurological disorders in terms of disease prevalence (age standardized) and years lived with the disease (YLD) in both sexes [1]. Headache disorders usually affect both young and older adults (40%), thus resulting in the loss of many working hours (absenteeism) and lower productivity (presenteeism) [2]. Among all headache disorders, migraine has the third highest prevalence of all medical illnesses. According to the World Health Organization (WHO), disability due to migraine is greater even than disability attributed to cardiovascular disorders [3,4,5,6].

Multiple Sclerosis (MS) is an inflammatory disorder of the central nervous system with a high prevalence, ranking sixth in frequency among neurological disorders. Not only MS as a primary disease but also comorbidities frequently associated with MS, affect the quality of life of these patients, resulting in reduced productivity, as measured in disability-adjusted life years (DALYs) [7].

A definite association between headache disorders, and specifically migraine and MS, is not yet proven. Recent studies evaluated the incidence of headache disorders to be up to 64% in MS patients [8,9,10,11]. Many hypotheses have been speculated about the pathophysiological mechanism of headache in MS patients and the various co-factors involved. Experimental cortical demyelination that accelerates cortical spreading depression, the presence of meningeal and cortical B-cell follicles, and the specific location of lesions attempt to explain headache incidence in MS patients [12,13,14,15]. Freedman and Gray, who have studied the presence of headache in patients with MS during an attack, showed that nearly half of the patients had brain stem involvement [16]. Epidemiological studies confirm the aforementioned results and imaging studies have indicated that midbrain/periaqueductal (PAG) MS lesions are associated with an increased incidence of headache [17,18,19]. PAG as well as other midbrain structures and their connections to the rostral ventromedial medulla and the dorsolateral pontomesencephalic tegmentum have been associated with the occurrence of headache, by decreasing the firing of the nociresponsive neurons of the dorsal horn [17,20]. Additionally, retrospective observational studies that included patients who underwent deep brain electrical stimulation showed that migraine-like attacks were generated via the stimulation of PAG [21,22]. Other locations that have also been related to headache are the substantia nigra, the red nucleus and the hypothalamus, all of which are linked to PAG by afferent and efferent signaling [17]. Among the co-factors, MS therapies are considered to play a role.

With the development of research regarding B-cell implications in the pathophysiology of MS, new depleting therapies that target either B-cells alone or both T- and B-cells have been introduced in MS clinical practice (Rituximab, Ocrelizumab, Ofatumumab, Cladribine and Ublituximab). Common adverse events of B-cell therapies usually include lymphopenia, susceptibility to infections and an increased incidence of malignancies. The majority of clinical trials focus on severe adverse events (SAEs), such as the aforementioned. Minor side effects such as headache and dizziness are usually not systematically reported and are often underrated, resulting in missing data.

B-cell therapies that have been used in MS as well as in many other diseases (e.g., lymphoma, rheumatoid arthritis, chronic lymphocytic leukemia, pemphigus, etc.), have been insufficiently related to headache as an adverse event. However, this finding occurred mostly as an Infuse Related Reaction (IRR), as reported by the Summary of Product Characteristics (SmPC) of each drug [23,24,25,26,27,28,29,30,31,32,33]. Headache was noted largely in the real-world evidence status and not in the pre-market clinical studies, resulting in missing data regarding pain characteristics that would allow classification.

Headache as an adverse event has been assessed, but not meticulously reported, in many clinical trials among the different available MS therapies. A recent meta-analysis, which included all studies on interferon-beta (INF-β) in MS [34], showed increased headache incidence over placebo in patients receiving INF-β (Relative Risk 1.16 [95% Confidence Interval 1.02–1.33], *p*-value = 0.02), who have reported de novo headaches, the aggravation of pre-existing headaches or change in the clinical features. INF-β was one of the most widely prescribed immunomodulatory MS treatment until now. According to the International Classification of Headache Disorders 3rd edition (ICHD-3) criteria [35], this type of headache is categorized as secondary (§ 8.1.10 Headache attributed to long-term use of non-headache medication) and studies speculate potential underlying pathogenetic mechanisms related to INF-β, as the headache attack has a close temporal relation to the subcutaneous injection of the drug. In vitro studies show that IFN-β influences the neuronal excitability in neocortical pyramidal neurons [36,37], which seems to play an important role in the pathogenesis of primary headaches [38]. Cytokine level changes (e.g., serum tumor necrosis factor alfa and IL-10) should also be evaluated, as high serum levels are reported in headache patients [37,39,40]. Patients receiving fingolimod in a FREEDOMS trial had an increased incidence of headache compared to placebo (26.6% vs. 23%, *p*-value = 0.007) [41]. Furthermore, fingolimod is associated with posterior reversible encephalopathy syndrome (PRES) and there are several cases that support a temporal correlation of fingolimod treatment initiation with new and persistent headaches [42,43]. A direct endothelial modulatory effect has been hypothesized by the authors as a mechanism of action [43,44,45,46].

A possible common denominator might be the increase in IL-10 levels. Munno et al. [39] showed that IL-10 levels were increased in patients during migraine attacks and subsequently decreased after sumatriptan treatment. Both INF-β and fingolimod result in an increase in IL-10 expression in circulating B-cells [47,48,49] and myeloid cells [50].

Taking the above into consideration together with the higher IL-10 levels produced by the reconstituted B-cells particularly following anti-CD20 treatment [50], we hypothesize that anti-B-cell therapies might thereby contribute to increased headache incidence in patients with MS.

However, to date, no previous studies exist that review the incidence of headache as an adverse event in MS patients receiving different anti-B-cell therapies. To provide additional evidence, we conducted a systematical review and meta-analysis to investigate the association of B-cell targeted therapies with headache incidence in MS patients.

## 2. Methods

This review was conducted in accordance with the Preferred Reporting Items for Systematic Review and Meta-analysis Protocols (PRISMA) statement [51,52,53]. The protocol was registered in the International Prospective Register of Systematic Reviews (PROSPERO; CRD42020199534). No institutional review board approval was required because all the study data were published previously and this study did not include individual patient data.

### 2.1. Eligibility Criteria, Literature Search and Study Selection

**Figure 1:**
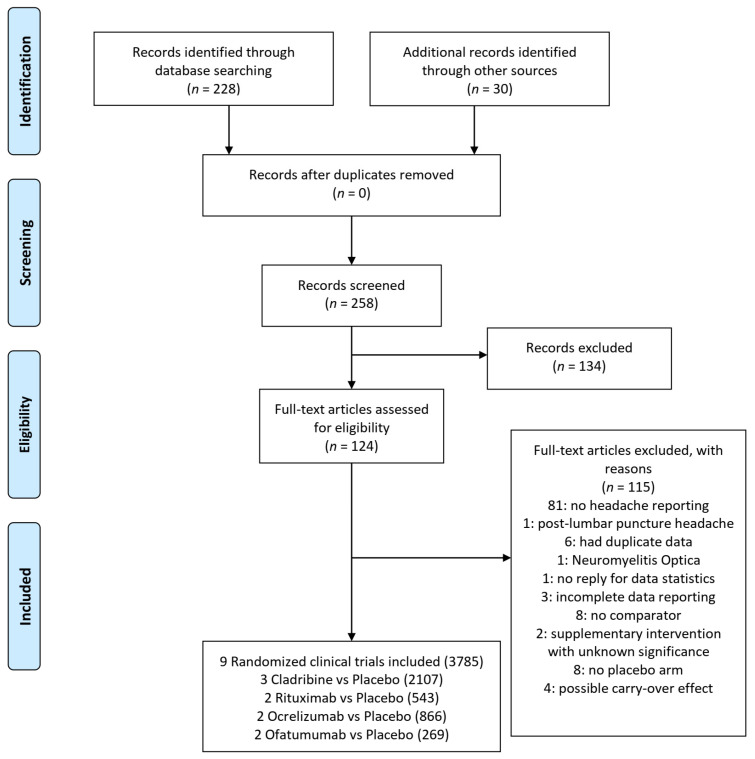
PRISMA Flow Diagram.

A systematic literature search was conducted through June 2020 to identify randomized placebo-controlled trials enrolling adult patients (>18 years of age) with Multiple Sclerosis treated with B-cell targeted therapies (Rituximab, Ocrelizumab, Cladribine, Ofatumumab and Ublituximab). The following electronic bibliographic databases were searched from inception until the 6th of June 2020 using a comprehensive search strategy: MEDLINE and PubMed (non-MEDLINE records only). ClinicalTrials.gov and EU Clinical Trials Register (clinicltrialsregister.eu) were also searched for all registered clinical trials and RCTs. The search strategy was structured according to the Peer Review of Electronic Search Strategies (PRESS) 2015 guidelines [54]. No limits were applied to sex or race. Only English language articles were considered. The full texts of articles identified as relevant during the title and abstract screening stage were obtained and reviewed. The comprehensive search strategy, detailed information about the inclusion and exclusion criteria and the PRISMA statement along with the screening/eligibility process are described in Figure 1 and in the Appendix BAppendix C and Appendix D and Supplement Table S1.

### 2.2. Outcomes

The primary outcome was the incidence in headache (all types) reported as an adverse event throughout the whole duration of the studies selected.

### 2.3. Data Extraction and Risk of Bias

Two reviewers (T.M. and M.B.) independently extracted individual study data and four reviewers (T.M., M.B., N.P., A.D. and A.L.) evaluated studies for the risk of bias using the Cochrane Risk of Bias tool for RCTs (ROB-II) [55]. The following domains of each of the primary studies were assessed: random sequence generation, allocation concealment, blinding of study participants, incomplete outcome data, selective reporting and other biases. Based on these domains, the overall risk of bias for each included study was assessed.

### 2.4. Statistical Analyses

We applied random-effects meta-analysis to Risk Ratio (RR) estimating the association of headache with the B-cell targeted treatment of Multiple Sclerosis. Random-effects models were a priori preferred over fixed-effects models due to the expected heterogeneity between studies with regard to intervention and outcome definition. The significance level for the overall effect was set at *p* < 0.05. Between-study heterogeneity was assessed by the I^2^ and the Cochran’s Q test; the significance level was defined by a *p* value < 0.05 or I^2^ > 50%. A sub-group analysis was performed for the different pharmacological interventions. All analyses were performed using R v. 4.0 (R foundation, Vienna, Austria) with the ‘metafor’ package [56,57,58].

## 3. Results

We identified 258 records that were eligible for inclusion, including 30 RCTs (Figure 1). The included trials evaluated four interventions (Rituximab, Ocrelizumab, Cladribine, Ofatumumab). There were no data available concerning headache as an adverse event in clinical trials of Ublituximab.

Summary of Study Retrieval and Identification for Meta-analysis according to PRISMA statement.

In total, nine studies [59,60,61,62,63,64,65,66,67] (3785 patients) were included in the final analysis, whose characteristics are shown in Table 1.

Cladribine (a synthetic purine nucleoside analog that inhibits DNA synthesis and ribonucleotide reductase) was assessed in three studies (2107 participants), Ocrelizumab (a humanized anti-CD20 monoclonal antibody) in two studies (866 participants), Ofatumumab (a fully human anti-CD20 monoclonal antibody) in two studies (269 participants) and Rituximab, a chimeric anti-CD20 monoclonal antibody, also in two studies (543 participants), as presented in Table 2.

**Figure 2:**
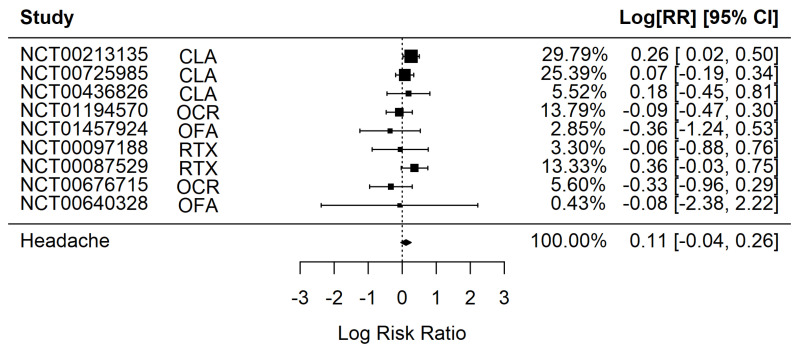
Forest plot for the association of headache with B-Cell modifying therapies. NCT: clinicaltrials.gov registration number, CLA: Cladribine, OCR: Ocrelizumab, RTX: Rituximab, OFA: Ofatumumab, RR: Risk Ratio, CI: Confidence Interval.

The presence of headache was not associated, in general, with B-cell depleting therapy (RR 1.12 [95% CI 0.96–1.30], *p*-value = 0.15, I^2^ = 9.32%, Q = 7.42, *p* = 0.492) (Figure 2).

**Figure 3:**
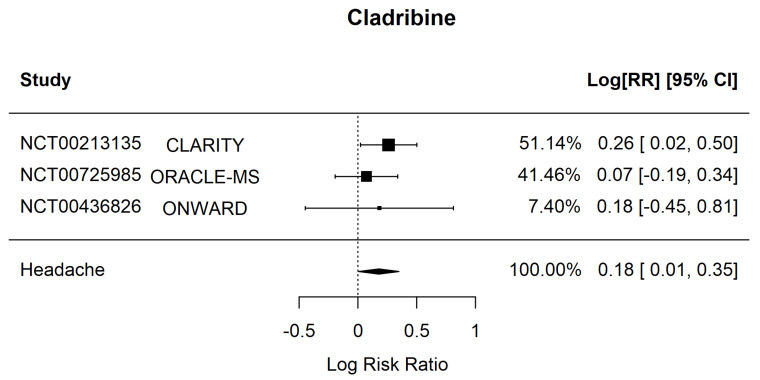
Forest plot of sub-group analysis for the association of headache with Cladribine treatment. NCT: clinicaltrials.gov registration number, RR: Risk Ratio, CI: Confidence Interval.

By extracting the Risk Ratio of headache incidence from the included randomized control trials for each drug, a statistically significant association between headache and treatment with Cladribine was uncovered (RR 1.20, [95% CI 1.006–1.42], *p*-value = 0.042, I^2^ = 0%, Q = 1.07, *p* = 0.586) (Table 2, Figure 3).

A sub-group analysis of the included studies for every monoclonal antibody against the B-cell line (i.e., Ofatumumab, Rituximab and Ocrelizumab) and for each drug separately showed no apparent connection between the development of headache and the different treatment options (Supplement Figures S1–S4).

According to ROB-II, all studies fall into the category of some concerns of bias. The main reason is the absence of a finalized pre-specified analysis plan before unblinding the outcome data that were available for headache assessment analysis. As a result, there were some concerns in the domain of bias in the selection of the reported result. In all other ROB-II domains there was low risk of bias (Supplement Figure S5 and S6).

Most of the headache-related data we present here were extracted from clinicaltrials.gov and were not available in the respective trial’s publications. There is a concern of possible publication bias, especially since headaches are considered secondary adverse effects, but this concern is minimized due to the obligatory reporting in clinicaltrials.gov. The analysis of our results indicates that it probably does not have a big effect in this case (Egger’s test *p*-value = 0.18, Supplement Figure S7).

## 4. Discussion

We performed a meta-analysis and compared the relative risk of headache in patients with MS under treatment with B-cell depletion therapies to those receiving placebo and found that B-cell targeted therapies are not associated overall with an increased risk of developing secondary headache. Furthermore, we conducted a Risk Ratio analysis separately for each drug included in the original analysis. No apparent correlation was found between the development of headache and monoclonal antibodies against B-cells (Supplement Figures S1–S4). Interestingly, an association of Cladribine and headache incidence was identified (Figure 3). As comparison between B-cell therapies and other MS treatments (head-to-head clinical trials) was unfeasible, due to cross-over studies and many confounding factors. A placebo comparator was a priori decided for the study analysis.

The route of administration plays an important role, especially when it involves parenteral administration, injection related adverse events and high frequency dosing regimen. B-cell therapy studies are more appropriate for investigating headache incidence, as they do not bear the risk of injection-associated headache, as opposed to interferons that are administered subcutaneously with a high frequency. Thus, the above results depict the active-drug’s action more, rather than the route of administration.

Although headache is among the commonly reported side effects of Cladribine, an induction mechanism has not been proposed so far. The modulation of nociception by perturbations introduced in purinergic signaling cascades may explain headaches. The contribution of purinergic signaling in pain conduction encompasses both vasomotor, neuronal and cortical processes, closely following the dissemination of purinergic receptors in each tissue [68]. Of note, the ATP-mediated migrainogenic activation of trigeminal nerves has been shown to be regulated by the calcitonin gene-related peptide (CGRP) [69]. Experimental models have furthermore determined that the purinergic modulation of nociception or the algogenic activation of the trigeminal ganglia may occur either directly, i.e., via activation of algogenic P2-calcium receptors [70], or indirectly by modulating the nitroxidergic system peripherally [71].

In addition, early studies of radioligand binding affinity demonstrated that Cladribine binds as an agonist to the adenosine receptor sub-types A_1_ and A_2A_ [72]. This may be relevant to the emergence of headache in Cladribine-treated patients, as A_2A_ receptor-mediated signaling is thought to be central to the pathophysiology of headaches [73]. Interestingly, A_1_ receptor mRNA and protein levels were found to be reduced in MS brain tissue [74] and A_1_ receptor deficiency increased proinflammatory responses and aggravated experimental allergic encephalomyelitis in mice [75]. This may suggest that transcriptional control or the transcript degradation of the A_1_ receptor gene is perturbed during MS-associated neuroinflammation. It is therefore conceivable that A_2A_ receptors are disproportionately expressed in the CNS of MS patients, resulting in excess A_2A_ receptor-mediated signaling upon treatment with Cladribine. A possible purinergic signaling cascade and disproportional expression of A_2A_ over A_1_ receptors, as mechanism of action, should be investigated through further studies.

This study is not without caveats. Limitations of this study include the heterogeneity of the drugs under investigation, the different categorization of headache disorders and MS criteria and variability in methods used. More specifically, the chronological spectrum of the included studies in the analysis ranges from 2008 to 2019. During this period, different diagnostic criteria for both MS and headache disorders were applied. Furthermore, all the included studies share in common that they report every type of headache together as one group, named in general as “head pain”, without sub-dividing into further categories such as migraine, TTH or other type of primary or secondary headache disorder.

As stated previously, minor adverse events such as headache were not the primary study interest of the clinical trials. For this reason, several studies have disclosed that the data were collected by a non-systematic assessment. Consequently, there were missing data in the clinical trials of our interest.

We should also note that even though all the treatments analyzed in this study have a reported direct or indirect B-cell mediated mechanism of action, they do not share similar pharmacodynamics. Even the three so called “anti-CD20 monoclonal antibodies” (Rituximab, Ocrelizumab, Ofatumumab), despite sharing the “same” target (B-cells that express CD20), differ in the exact neuroimmunomodulatory effects. Moreover, Cladribine is a synthetic purine nucleoside analog that inhibits DNA synthesis and ribonucleotide reductase. Once inside the cell, Cladribine is activated mostly in lymphocytes, after being triphosphorylated by the enzyme deoxyadenosine kinase (dCK). Activated, the triphosphorylated Cladribine is incorporated into mitochondrial and nuclear DNA, which triggers apoptosis [76]. Due to the extremely specific ratio of dCK to 5′-NTase needed to activate and accumulate enough Cladribine to induce apoptosis, only lymphocytes are uniquely vulnerable [77]. Within the lymphocyte pool, Cladribine targets B-cells more than T cells. The CD3+ T cells remain suppressed longer than the CD19+ B-cells, and the CD4+ cells are affected more than the CD8+ cells.

Overall, the shift to B-cell therapies in the management of MS provided more and better tolerated treatment options. However, as newly introduced players emerge in the field of MS therapeutics, increased pharmacovigilance is needed. With every new treatment introduced, the expectation of a more individualized, targeted and better tolerated medical care raises.

## 5. Conclusions

In this study, we show for the first time that contrary to the rationale of being expected, B-cell targeted MS therapies are not significantly associated with the incidence of headache as an adverse effect. However, a statistically significant association (RR 1.20, [95% CI 1.006–1.42], *p*-value = 0.042) between Cladribine and an increased incidence of headache was observed. Physicians, apart from severe adverse events (SAEs), such as lymphopenia, hepatotoxicity, carcinogenesis and opportunistic infections, also need to keep in mind those minor adverse effects to achieve treatment adherence through patient education. Further research is needed to elucidate the pathogenetic mechanism of headache induction in B-cell targeted MS therapies, as well as to identify headache prevention strategies.

## Figures and Tables

**Table 1:** Main characteristics of the included trials.

NCT	Trial Name	Sponsor	Intervention	Age, Mean, y (SD)	# of Participants Included in the Safety Population Analysis	# Treated with H/Treated	# Placebo with H/Placebo
NCT00213135 [65]	CLARITY	EMD Serono	CLA	38.6 (10.0)	1319	198/884	75/435
NCT00725985 [61]	ORACLE-MS	EMD Serono	CLA	31.9 (8.7)	616	122/410	57/206
NCT00436826 [64]	ONWARD	EMD Serono	CLA	38.9 (10.2)	172	31/124	10/48
NCT01194570 [60]	ORATORIO	Hoffmann-La Roche	OCR	44.6 (8.0)	702	68/486	33/216
NCT01457924 [59]	MIRROR	GlaxoSmithKline	OFA	37.2 (9.36)	231	12/164	7/67
NCT00097188 [66]	HERMES	Genentech, Inc.	RTX	40	104	13/69	7/35
NCT00087529 [67]	OLYMPUS	Genentech, Inc.	RTX	49.9 (8.90)	439	77/292	27/147
NCT00676715 [62]	-	Genentech, Inc.	OCR	37.6 (8.8)	164	19/110	13/54
NCT00640328 [63]	OMS115102	GlaxoSmithKline	OFA	36.3 (7.9)	38	2/26	1/12

NCT: clinicaltrails.gov registration number, #: number, CLA: Cladribine, OCR: Ocrelizumab, RTX: Rituximab, OFA: Ofatumumab, CLARITY: A Safety and Efficacy Study of Oral Cladribine in Subjects With Relapsing-remitting Multiple Sclerosis (RRMS), ORACLE-MS: Oral Cladribine in Early Multiple Sclerosis (MS), ONWARD: A Phase 2 Study of Cladribine Add-on to Interferon-beta (IFN-beta) Therapy in Multiple Sclerosis (MS) Subjects With Active Disease, ORATORIO: A Study of Ocrelizumab in Participants With Primary Progressive Multiple Sclerosis, MIRROR: Ofatumumab Subcutaneous Administration in Subjects With Relapsing-Remitting Multiple Sclerosis, HERMES: A Study to Evaluate Rituximab in Adults With Relapsing Remitting Multiple Sclerosis, OLYMPUS: A Study to Evaluate the Safety and Efficacy of Rituximab in Adults With Primary Progressive Multiple Sclerosis, OMS115102: Ofatumumab Dose-finding in Relapsing Remitting Multiple Sclerosis (RRMS) Patients.

**Table 2:** Main characteristics of study results by intervention.

Intervention	# of Trials	# of Patients	RR	95% CI	*p*-Value	I^2^
Cladribine	3	2107	1.20	1.006–1.42	0.042	0%
Ocrelizumab	2	866	0.857	0.93–1.89	0.115	0%
Ofatumumab	2	269	0.728	0.33–1.66	0.448	0%
Rituximab	2	543	1.33	0.62–1.19	0.354	0%
Total	9	3785	1.12	0.96–1.30	0.15	9.32%

#: Number, RR: Risk Ratio for the incidence of headache, CI: Confidence Interval, I^2^ Study Heterogeneity.

## Data Availability

The data that support the findings of this study are available in Clinicaltrials.gov at https://clinicaltrials.gov/ (assessed on 6 June 2020).

## Supplementary Materials

The following supporting information can be downloaded at: https://www.mdpi.com/article/10.3390/jpm12091474/s1.

## Appendix A

**Table:**

Name	Location	Role	Contribution
Theodoros Mavridis	First Department of Neurology, Eginition Hospital, Medical School, National and Kapodistrian University of Athens, Athens, Greece	Author	Design and conceptualized study; acquisition of data; interpreted the data; drafted the manuscript for intellectual content
Nikolaos Papagiannakis	First Department of Neurology, Eginition Hospital, Medical School, National and Kapodistrian University of Athens, Athens, Greece	Author	Acquisition of data; methodology; curation and interpretation of data; formal analysis; visualization data; investigation; review and editing
Marianthi Breza	First Department of Neurology, Eginition Hospital, Medical School, National and Kapodistrian University of Athens, Athens, Greece	Author	Acquisition of data; methodology; curation and interpretation of data; formal analysis; visualization data; investigation; review and editing
Georgios Vavougios	Scientific Research Associate, Neuroimmunology Laboratory, Department of Neurology, Athens Naval Hospital, Athens, Greece	Author	Acquisition of data; methodology; curation and interpretation of data; formal analysis; visualization data; investigation; review and editing
Kostas Patas	First Department of Neurology, Eginition Hospital, Medical School, National and Kapodistrian University of Athens, Athens, Greece	Author	Acquisition of data; methodology; curation and interpretation of data; formal analysis; visualization data; investigation; review and editing
Ariadne Daponte	First Department of Neurology, Eginition Hospital, Medical School, National and Kapodistrian University of Athens, Athens, Greece	Author	Acquisition of data; methodology; curation and interpretation of data; formal analysis; visualization data; investigation; review and editing
Achilleas Laskaratos	National and Kapodistrian University of Athens, Medical School, Athens, Greece	Author	Acquisition of data; revised the manuscript for intellectual content
Paraschos Archontakis-Barakakis	Northeast Internal Medicine Associates, LaGrange, Indiana, USA.	Author	Formal analysis; revised the manuscript for intellectual content; editing
Ioannis Pantazopoulos	Department of Emergency Medicine, Faculty of Medicine, University of Thessaly, Biopolis, 41500, Larissa, Greece	Author	Revised the manuscript for intellectual content; supervision of the entire process; project administration
Dimos D. Mitsikostas	First Department of Neurology, Eginition Hospital, Medical School, National and Kapodistrian University of Athens, Athens, Greece	Author	Revised the manuscript for intellectual content; supervision of the entire process; project administration

## Appendix B. Systematic Review Search Strategy

Database searching:

**Table:**

Name of Database (incl. Interface)	Why Is It Relevant (to the Topic)?	Does It Offer a Controlled Vocabulary/Subject Headings?	Do You Have Access to This Database?
MEDLINE (PubMed)	MEDLINE is the U.S. National Library of Medicine^®^ (NLM) premier bibliographic database that contains more than 26 million references to journal articles in life sciences with a concentration on biomedicine.	Yes, MeSH terms	Yes

Structure of the search strategy:

**Table:**

Concept	Importance
Rituximab	High—search terms for this have to be included
Ocrelizumab	High—search terms for this have to be included
Ofatumumab	High—search terms for this have to be included
Ublituximab	High—search terms for this have to be included
Cladribine	High—search terms for this have to be included
Multiple Sclerosis	High—search terms for this have to be included
Clinical Trials	High—search terms for this have to be included

Supplementary searches: What other information sources are relevant for to this topic? Choose at least two and explain why you would use them.

**Table:**

Name of Source/Technique	Why Is It Relevant (to the Topic)?
Reference list	It may find studies using the “snowball” technique, that may not appear in the first search results.
Clinicaltrials.gov	It may find studies that may not appear in the first search results.
Clinicaltrialsregister.eu	It can help identify more studies than clinicaltrials.gov on this specific subject.

### Appendix B.1. Pubmed/MEDLINE Search Strategy

Time of search: Last search on 6 June 2020.

### Appendix B.2. Boolean Search String

((((((rituximab) OR ocrelizumab) OR ofatumumab) OR cladribine) OR ublituximab) AND multiple sclerosis) AND clinical trial

### Appendix B.3. Actual MeSH Term Search

(((((((“rituximab”[MeSH Terms] OR “rituximab”[All Fields]) OR “rituximab s”[All Fields]) OR (“ocrelizumab”[Supplementary Concept] OR “ocrelizumab”[All Fields])) OR (“ofatumumab”[Supplementary Concept] OR “ofatumumab”[All Fields])) OR ((“cladribin”[All Fields] OR “cladribine”[MeSH Terms]) OR “cladribine”[All Fields])) OR (“ublituximab”[Supplementary Concept] OR “ublituximab”[All Fields])) AND ((“multiple sclerosis”[MeSH Terms] OR (“multiple”[All Fields] AND “sclerosis”[All Fields])) OR “multiple sclerosis”[All Fields])) AND ((“clinical trial”[Publication Type] OR “clinical trials as topic”[MeSH Terms]) OR “clinical trial”[All Fields])

Results: 228 entries.

### Appendix B.4. Additional/Supplementary Search

Search through reference lists and clinicaltrials.gov (“snowball technique”).

Results: 30 clinical trials.

## Appendix C. Inclusion/Exclusion Criteria

**Table:**

Inclusion Criteria	Exclusion Criteria
Study Design ○ Randomized placebo-controlled clinical trials (RCTs) Population ○ Adult patients (≥18 years) ○ Patients with definite MS diagnosis Intervention (B-cell targeted therapy) ○ Rituximab ○ Ocrelizumab ○ Ofatumumab ○ Cladribine ○ Ublituximab Comparator ○ Placebo Outcome ○ Headache reported as an adverse event (AE) Language ○ English	Study Design ○ Reviews ○ Observational trials ○ Case/Reports series ○ Cross-sectional trials ○ No-placebo controlled trials ○ Animal studies Population ○ Children and Adolescents (<18 years) ○ Patients with indefinite MS diagnosis ○ Patients with other demyelinating syndromes (e.g., Neuromyelitis Optica Spectrum Disorder, MOG syndrome) Comparator ○ Other than placebo (e.g., other active drug) Outcome ○ No headache reported as an adverse event (AE) Language ○ Other than English language

### Additional Information Regarding Exclusion Criteria

The monoclonal antibody against the surface glycoprotein CD52, alemtuzumab, was not included to the initial search. Alemtuzumab is a humanized anti-CD52 IgG1 monoclonal antibody that depletes CD52-expressing cells from the circulation. Data derived from experimental and clinical trials and long-term observational studies indicate that alemtuzumab induces a marked immunosuppression related to the depletion of circulating T and B lymphocytes (non-selective immune reconstitution). While it targets both T and B-cells, it induces a deep T cell and a rather milder B-cell depletion, in contrast to the Cladribine that was included. Thus, its non-selectivity and the preference towards the T cell linage were the main reasons for excluding it.

## Appendix D. PRISMA Statement and Screening/Eligibility Strategy

Preferred Reporting Items for Systematic Reviews and Meta-Analyses (PRISMA) guidelines for systematic reviews were applied in form of the PRISMA 2009 checklist (Table S1) and flow diagram (Figure S1) [51,52].

The first screening of the 258 reports resulted in 124 full articles that were assessed for eligibility. According to the above inclusion and exclusion criteria, 115 full-text articles and registered clinical trials were excluded with reasoning. The screening and eligibility phase was blindly conducted by two individual researchers (T.M. and M.B.). For the interrater reliability of selection, a test was performed on older data collection studies by calculating the statistical k (>90%). In case of a disagreement (<5%), two other researchers (G.V. and A.L.) carried out an audit of these studies based on the same inclusion and exclusion criteria. During the eligibility process, studies without provided results of adverse events were identified and raw data from clinicaltrials.gov and clinicaltrialsregister.eu were collected. An attempt was made to communicate to the corresponding author of one study meeting the above criteria without success. We subsequently further hand searched the reference lists of eligible articles and relevant reviews (the “snowball” procedure). All studies were evaluated for potential population overlap based on geographical setting, recruitment periods and same registration numbers. In case of overlapping populations, we retained the study with the largest sample size. In total, nine studies were finally included in the qualitative synthesis of this systematical review and subsequent meta-analysis. Our primary outcome of interest was all-cause headache as a side effect, as defined by standard clinical criteria.
