# Supplementary material for: B-Cell Targeted Therapies in Patients with Multiple Sclerosis and Incidence of Headache: A Systematic Review and Meta-Analysis
Source: J Pers Med. 2022 Sep 8;12(9):1474. doi: 10.3390/jpm12091474 (PMC9504525; doi:10.3390/jpm12091474)
Supplement: Supplementary file 1 [file jpm-12-01474-s001.zip › jpm-1883207-supplementary.pdf]

**Table S1. PRISMA Checklist.**

| Section/topic | # | Checklist item | Reported on page # |
|---|---|---|---|
| **TITLE** | | | |
| Title | 1 | Identify the report as a systematic review, meta-analysis or both. | 1 |
| **ABSTRACT** | | | |
| Structured summary | 2 | Provide a structured summary including, as applicable: background; objectives; data sources; study eligibility criteria, participants and interventions; study appraisal and synthesis methods; results; limitations; conclusions and implications of key findings; systematic review registration number. | 2-4 |
| **INTRODUCTION** | | | |
| Rationale | 3 | Describe the rationale for the review in the context of what is already known. | 4-6 |
| Objectives | 4 | Provide an explicit statement of the questions being addressed with reference to participants, interventions, comparisons, outcomes and study design (PICOS). | 6 |
| **METHODS** | | | |
| Protocol and registration | 5 | Indicate if a review protocol exists, if and where it can be accessed (e.g., Web address) and, if available, provide registration information including registration number. | 7 |
| Eligibility criteria | 6 | Specify study characteristics (e.g., PICOS, length of follow-up) and report characteristics (e.g., years considered, language, publication status) used as criteria for eligibility, given the rationale. | 7 |
| Information sources | 7 | Describe all information sources (e.g., databases with dates of coverage, contact with study authors to identify additional studies) in the search and the date last searched. | 7 |
| Search | 8 | Present full electronic search strategy for at least one database, including any limits used, such that it could be repeated. | Supplementary 1-2 |
| Study selection | 9 | State the process for selecting studies (i.e., screening, eligibility, included in systematic review and, if applicable, included in the meta-analysis). | Manuscript 7, Supplementary 2-4 |
| Data collection process | 10 | Describe the method of data extraction from reports (e.g., piloted forms, independently, in duplicate) and any processes for obtaining and confirming data from investigators. | 8 |
| Data items | 11 | List and define all variables for which data were sought (e.g., PICOS, funding sources) and any assumptions and simplifications made. | 8 |
| Risk of bias in individual studies | 12 | Describe the methods used for assessing the risk of bias of individual studies (including specification of whether this was done at the study or outcome level) and how this information is to be used in any data synthesis. | 8 |
| Summary measures | 13 | State the principal summary measures (e.g., risk ratio, difference in means). | 8-9 |
| Synthesis of results | 14 | Describe the methods of handling data and combining the results of studies, if done, including measures of consistency (e.g., $I^2$) for each meta-analysis. | 8-9 |

| Section/topic | # | Checklist item | Reported on page # |
|---|---|---|---|
| Risk of bias across studies | 15 | Specify any assessment of risk of bias that may affect the cumulative evidence (e.g., publication bias, selective reporting within studies). | Supplementary 10-12 |
| Additional analyses | 16 | Describe methods of additional analyses (e.g., sensitivity or sub-group analyses, meta-regression), if done, indicating which were pre-specified. | - |
| **RESULTS** | | | |
| Study selection | 17 | Give the number of studies screened, assessed for eligibility and included in the review, with reasons for exclusions at each stage, ideally with a flow diagram. | 9-10 |
| Study characteristics | 18 | For each study, present characteristics for which data were extracted (e.g., study size, PICOS, follow-up period) and provide the citations. | 9-10 |
| Risk of bias within studies | 19 | Present data on the risk of bias of each study and, if available, any outcome level assessment (see item 12). | Supplementary 10-12 |
| Results of individual studies | 20 | For all outcomes considered (benefits or harms), present, for each study: (a) simple summary data for each intervention group and (b) effect estimates and confidence intervals, ideally with a forest plot. | 9-10 |
| Synthesis of results | 21 | Present the results of each meta-analysis done, including confidence intervals and measures of consistency. | 9-10 |
| Risk of bias across studies | 22 | Present the results of any assessment of risk of bias across studies (see Item 15). | Supplementary 10-12 |
| Additional analysis | 23 | Give the results of additional analyses, if done (e.g., sensitivity or sub-group analyses, meta-regression [see Item 16]). | - |
| **DISCUSSION** | | | |
| Summary of evidence | 24 | Summarize the main findings including the strength of the evidence for each main outcome; consider their relevance to key groups (e.g., healthcare providers, users and policy makers). | 10-13 |
| Limitations | 25 | Discuss limitations at the study and outcome level (e.g., risk of bias) and at review level (e.g., incomplete retrieval of identified research, reporting bias). | 12-13 |
| Conclusions | 26 | Provide a general interpretation of the results in the context of other evidence and implications for future research. | 13-14 |
| **FUNDING** | | | |
| Funding | 27 | Describe sources of funding for the systematic review and other support (e.g., the supply of data); the role of the funders in the systematic review. | 18 |

*From:* Moher D, Liberati A, Tetzlaff J, Altman DG, The PRISMA Group (2009). Preferred Reporting Items for Systematic Reviews and Meta-Analyses: The PRISMA Statement. PLoS Med 6(7): e1000097. doi:10.1371/journal.pmed1000097

For more information, visit: www.prisma-statement.org.

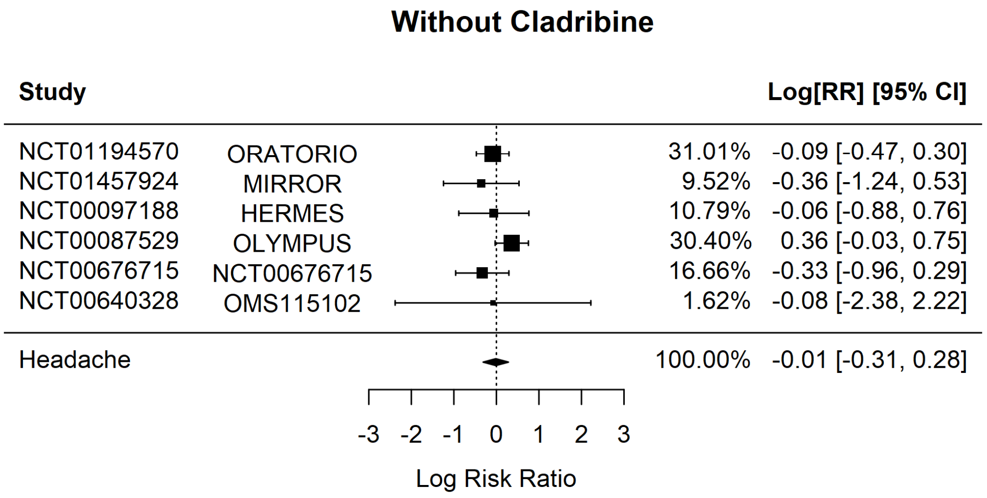


**Figure S1. Forest plot of sub-group analysis for the association of headache with B-cell targeted monoclonal antibody treatment.**

**NCT: clinicaltrials.gov registration number, RR: Risk Ratio, CI: Confidence Interval**

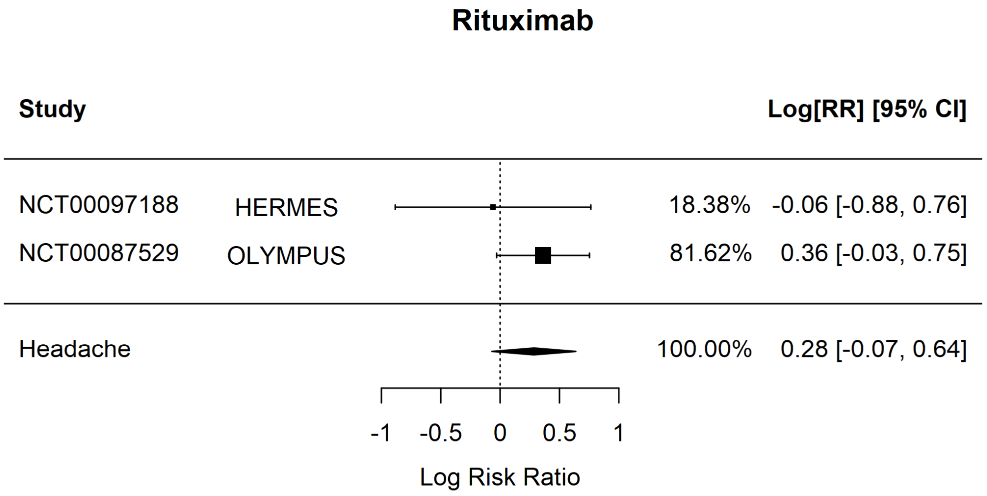


**Figure S2. Forest plot of sub-group analysis for the association of headache with Rituximab.**

**NCT: clinicaltrials.gov registration number, RR: Risk Ratio, CI: Confidence Interval**

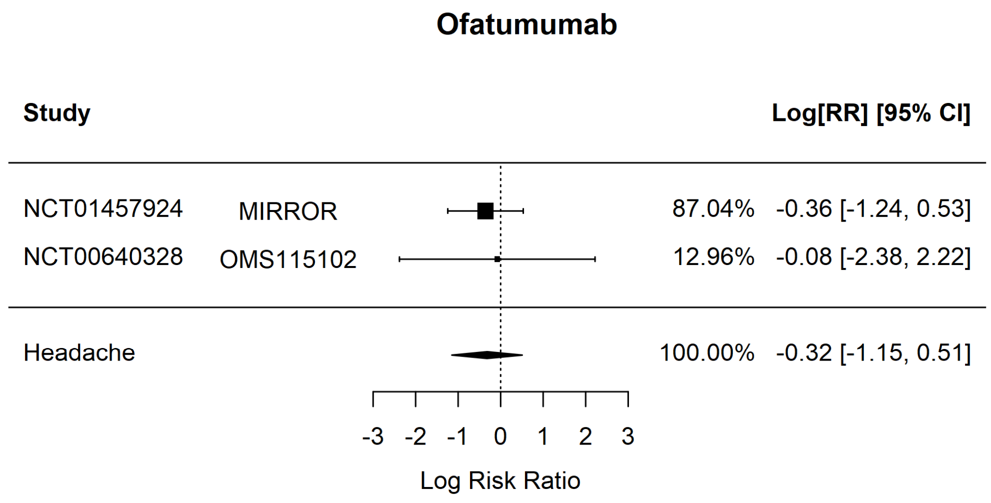


**Figure S3. Forest plot of sub-group analysis for the association of headache with Ofatumumab.**

**NCT: clinicaltrials.gov registration number, RR: Risk Ratio, CI: Confidence Interval**

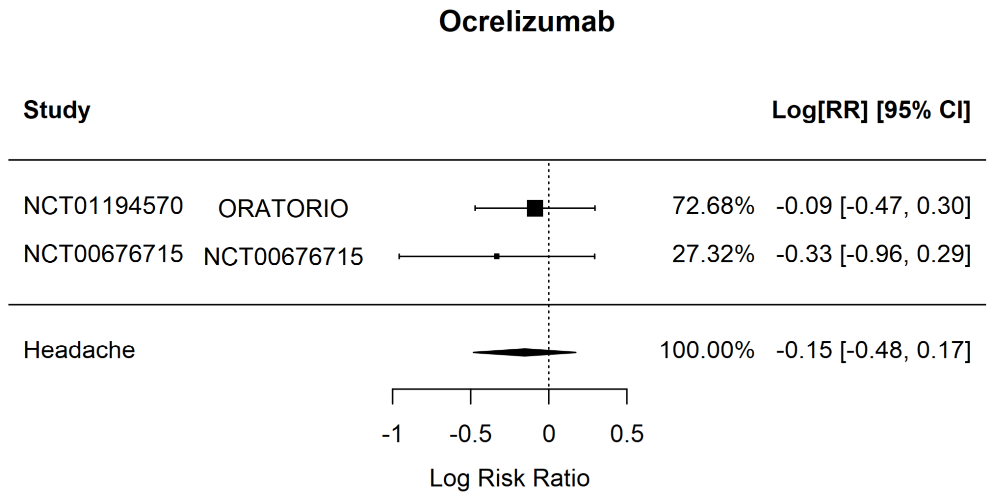


**Figure S4. Forest plot of sub-group analysis for the association of headache with Ocrelizumab.**

**NCT: clinicaltrials.gov registration number, RR: Risk Ratio, CI: Confidence Interval**

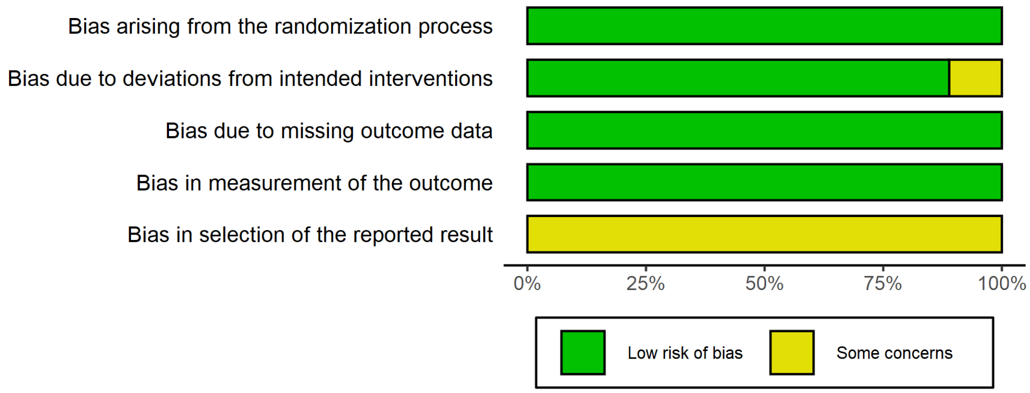


**Figure S5. Risk of bias of the included studies according to Cochrane ROB-II evaluation tool for randomized trials.**

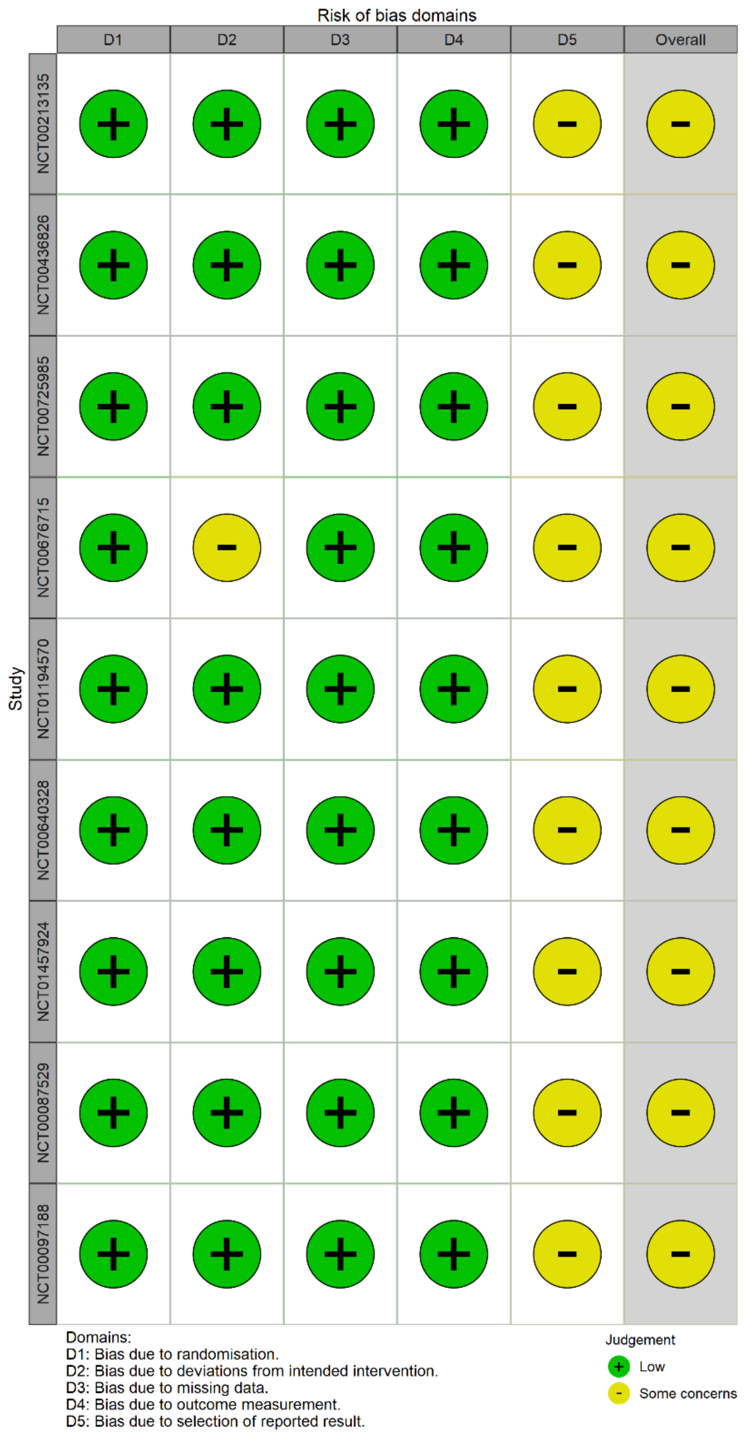


**Figure S6. Traffic Light plot according to ROB-2 tool of the included studies.**

**NCT: clinicaltrials.gov registration number**

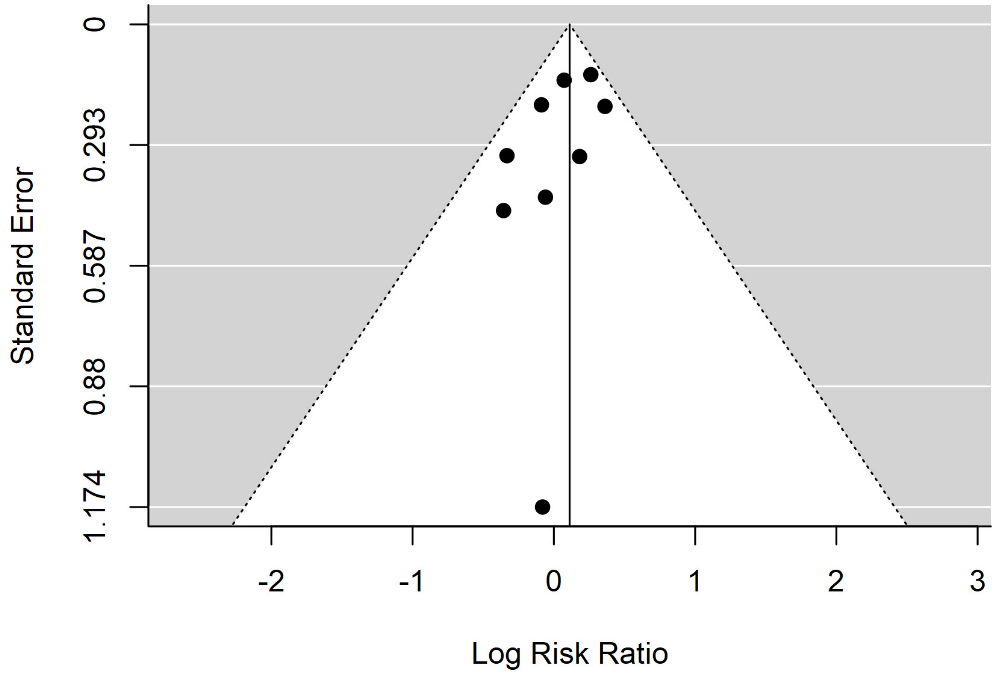


**Figure S7. Funnel plot for the presence of publication bias of the included studies.**
